# Red Cell Distribution Width Upon Hospital Admission Predicts Short-Term Mortality in Hospitalized Patients With COVID-19: A Single-Center Experience

**DOI:** 10.3389/fmed.2021.652707

**Published:** 2021-03-18

**Authors:** Christoph C. Kaufmann, Amro Ahmed, Ulrich Brunner, Bernhard Jäger, Gabriele Aicher, Susanne Equiluz-Bruck, Alexander O. Spiel, Georg-Christian Funk, Michael Gschwantler, Peter Fasching, Kurt Huber

**Affiliations:** ^1^Third Medical Department With Cardiology and Intensive Care Medicine, Wilhelminenhospital, Vienna, Austria; ^2^Department of Laboratory Medicine, Wilhelminenhospital, Vienna, Austria; ^3^Department of Hospital Hygiene, Wilhelminenhospital, Vienna, Austria; ^4^Department of Emergency Medicine, Wilhelminenhospital, Vienna, Austria; ^5^Karl-Landsteiner-Institute for Lung Research and Pulmonary Oncology, Wilhelminenhospital, Vienna, Austria; ^6^Department of Gastroenterology and Hepatology, Wilhelminenhospital, Vienna, Austria; ^7^Department of Endocrinology and Rheumatology, Wilhelminenhospital, Vienna, Austria; ^8^Sigmund Freud University, Medical School, Vienna, Austria

**Keywords:** red cell distribution width, COVID-19, short-term mortality, prognosis, pre-COVID-19

## Abstract

**Background:** Coronavirus disease (COVID-19) was first described at the end of 2019 in China and has since spread across the globe. Red cell distribution width (RDW) is a potent prognostic marker in several medical conditions and has recently been suggested to be of prognostic value in COVID-19.

**Methods:** This retrospective, observational study of consecutive patients with COVID-19 was conducted from March 12, 2020 to December 4, 2020 in the Wilhelminenhospital, Vienna, Austria. RDWlevels on admission were collected and tested for their predictive value of 28-day mortality.

**Results:** A total of 423 eligible patients with COVID-19 were included in the final analyses and 15.4% died within 28 days (*n* = 65). Median levels of RDWwere significantly higher in non-survivors compared to survivors [14.6% (IQR, 13.7–16.3) vs. 13.4% (IQR, 12.7– 14.4), *P* < 0.001]. Increased RDW was a significant predictor of 28-day mortality [crude odds ratio (OR) 1.717, 95% confidence interval (CI) 1.462–2.017; *P* = < 0.001], independent of clinical confounders, comorbidities and established prognostic markers of COVID-19 (adjusted OR of the final model 1.368, 95% CI 1.126–1.662; *P* = 0.002). This association remained consistent upon sub-group analysis. Our study data also demonstrate that RDW levels upon admission for COVID-19 were similar to previously recorded, non-COVID-19 associated RDW levels [14.2% (IQR, 13.3–15.7) vs. 14.0% [IQR, 13.2–15.1]; *P* = 0.187].

**Conclusions:** In this population, RDWwas a significant, independent prognostic marker of short-term mortality in patients with COVID-19.

## Key Takeaways

- The outbreak of coronavirus disease (COVID-19) originated late December, 2019, in Wuhan, China and has since spread rapidly across the globe causing an international pandemic. Several laboratory biomarkers have been established as predictors of mortality in patients with COVID-19.
- Red Cell Distribution Width (RDW) is a laboratory test used to measure the heterogeneity of erythrocyte volumes and assess the variation in size and form of circulating erythrocytes. Several studies identified RDW as a powerful, prognostic marker in a variety of medical conditions and it has recently been suggested to be of prognostic value in COVID-19.
- Our study finds that higher RDW values upon hospital admission were associated with a statistically significant increased risk of death from COVID-19 even after adjustment for clinical confounders, comorbidities and established prognostic, laboratory markers in COVID-19. We also found that RDWlevels upon admission for acute COVID-19 were comparable to previously recorded levels of RDW before SARS-CoV-2 infection.
- RDW may be used as an early marker of outcome in patients with COVID-19 and hence guide appropriate work-up and management of these patients.

## Introduction

The outbreak of coronavirus disease (COVID-19) originated late December, 2019, in Wuhan, China and has since spread rapidly across the globe causing an international pandemic. COVID-19 is caused by a single-stranded enveloped RNA virus called Severe Acute Respiratory Syndrome Coronarvirus-2 (SARS-CoV-2) (1). While most patients experience mild illness without significant respiratory manifestations, approximately 19% of patients have a severe disease course with reported case fatality rates of up to 49% among critically ill cases in China (2). Several laboratory markers have been identified to be of prognostic value in COVID-19, including lactate dehydrogenase (LDH), blood urea nitrogen (BUN) and neutrophil to lymphocyte ratio (NLR) among others (3–5). As of January 9, 2021 a total of 376.993 COVID-19 cases with 6.687 fatal outcomes were reported in Austria (6).

Red Cell Distribution Width (RDW) is a laboratory test used to measure the heterogeneity of erythrocyte volumes (anisocytosis) and assess the variation in size and form of circulating erythrocytes. Increased levels of RDW have emerged as a powerful, negative prognostic marker in several medical conditions, including inflammatory, pulmonary and cardiovascular disease (7–11). In patients with pneumonia, specifically, RDW was associated with both short- and long-term mortality in multiple studies (12–15). Recently RDW has also been suggested to be of prognostic value in patients with COVID-19 (16–20). Most notably, Foy et al. found a significantly increased mortality risk in patients with elevated RDW at the time of hospital admission with a hazard ratio of 2.01 when using a cut-off of 14.5% (21).

We sought to further test the hypothesis of increased short-term mortality in patients with higher levels of RDW in hospitalized patients with COVID-19. Additionally, we aimed to gather information on the temporal course of RDW levels in the context of COVID-19 by assessing levels of RDW prior to COVID-19 infection and by comparing RDW levels according to time to symptom onset.

## Materials and Methods

### Study Design and Participants

A total of 423 consecutive patients with laboratory confirmed SARS-CoV-2 infection were included in our retrospective single-center study. All patients presented to the emergency department and were admitted for in-hospital care between March 12, 2020 to December 4, 2020 at different wards of our institution (Supplementary Figure 1). The diagnosis of COVID-19 was made according to the WHO interim guidance and confirmed by PCR proven RNA detection of SARS-CoV-2 on nasal and/or pharyngeal swabs. Only patients 18 years or older were included for retrospective data collection. Our study was approved by the local ethics committee of the city of Vienna (EK 20-110-VK) and complies with the Declaration of Helsinki and the International Conference on Harmonization Guidelines for Good Clinical Practice. The authors had full access to the data and take responsibility for its integrity.

Demographic data, clinical features, laboratory results and medical history were obtained from patient records on admission. Follow-up data was collected through the patient record system of our institution until January 1, 2021. RDW is reported in percent as the coefficient of variation of red blood cell volume. The normal range for RDW in our laboratory is 11.5–14.5%. Patients were grouped by survival status 28 days after admission and by quartiles of RDW. The following laboratory data were recorded and compared among the groups: RDW, white blood cells, neutrophils, lymphocytes, c-reactive protein (CRP), red blood cell count, platelets, hemoglobin, mean corpuscular volume (MCV), creatinine, BUN, sodium, potassium, LDH and NLR.

Cardiovascular disease was defined by a history of coronary artery disease or heart failure. Chronic pulmonary disease was defined by a history of chronic obstructive pulmonary disease, asthma bronchiale or obstructive sleep apnea. Malignancy was defined as a history of solid tumors or hematological malignancy. All co-morbidities were defined at the discretion of the treating physician.

The primary outcome of our study was short-term mortality, defined as 28-day mortality. The prognostic impact of RDW upon hospital admission was also tested across several sub-groups, including age, gender, arterial hypertension, history of cardiovascular disease, anemia, levels of CRP and creatinine. We assessed pre-COVID-19 levels of RDW, hemoglobin and CRP in those patients with available blood drawings within 14 days and 2 years prior to admission for SARS-CoV-2 infection. Additionally, we compared levels of RDW according to time to symptom onset.

### Statistical Analysis

Continuous data are reported as median and interquartile range (IQR), and categorical data are expressed as frequency and percentage. Mann-Whitney U-test and Pearson's chi-squared tests were used to compare continuous and categorical data between survivors and non-survivors. Kruskal–Wallis test and Pearson's chi-squared tests were used to compare baseline characteristics and laboratory results across quartiles of RDW.

Univariable and multivariable cox regression analysis was performed to determine the prognostic impact of RDW on 28-day mortality with associations expressed as hazard ratios (HR) and 95% confidence intervals (CI). Laboratory markers, included in the regression models, were log-transformed prior to statistical analysis to improve normality (except for NLR). RDW was standardized by subtracting the mean and dividing by the standard deviation to improve visualization of results. The prognostic impact of RDW was also assessed as a binary variable at a cut-off of 14.5%. Pre-COVID-19 levels of RDW, hemoglobin and CRP were compared to levels at admission using Wilcoxon signed rank test. RDW levels according to symptom onset were compared using Kruskal-Wallis test.

All statistical analyses were performed using SPSS 26.0 (SPSS Inc., Chicago, IL, USA) and a two-sided *P*-value < 0.05 was required for statistical significance. Graphics were generated using GraphPad Prism 9.0 (GraphPad Software, Inc., San Diego, CA).

## Results

### Clinical Characteristics and Laboratory Findings Stratified by Survival Status

A total of 423 patients with COVID-19 were included in the study, of whom 65 died within 28 days (15.4%). Levels of RDW upon hospital admission were significantly higher in non-survivors compared to survivors [14.6% (IQR, 13.7–16.3) vs. 13.4% (IQR, 12.7–14.4)]. Non-survivors were also older (mean age of 81 ± 10.3 years vs. 65 ± 16.7 years) and more frequently had a history of arterial hypertension, cardiovascular disease, chronic pulmonary disease and/or chronic kidney disease. While dyspnea was more common among non-survivors, symptoms of gastrointestinal disease were less frequently reported. There was no difference in sex, history of diabetes mellitus or history of malignancy. Significantly higher levels of white blood cells, neutrophil to lymphocyte ratio, CRP, creatinine, BUN and LDH were observed in non-survivors, while levels of hemoglobin and red blood cells were significantly lower (Table 1).

**Table 1:** Baseline characteristics of the study population stratified by survival status.

**Characteristics**	**Survivors** ***n* = 358**	**Non-survivors** ***n* = 65**	***P*-value**
**Baseline characteristics**			
Age, years *(mean ± SD)*	65 ± 16.7	81 ± 10.3	** <0.001**
Male sex	204 (57.0%)	40 (57.7%)	0.495
Arterial hypertension	212 (59.2%)	50 (76.9%)	**0.007**
Diabetes mellitus	113 (31.6)	23 (35.4%)	0.545
Cardiovascular disease	76 (21.2%)	36 (55.4%)	** <0.001**
Chronic pulmonary disease	42 (11.7%)	15 (23.1%)	**0.014**
Chronic kidney disease	56 (15.7%)	20 (30.8%)	**0.004**
History of malignancy	42 (11.7%)	13 (20.0%)	0.069
**Signs and symptoms**			
Fever	248 (69.3%)	42 (64.6%)	0.457
Coughing	177 (49.4%)	31 (47.7%)	0.795
Dyspnea	189 (52.8%)	46 (70.8%)	**0.007**
Gastrointestinal symptoms	85 (23.7%)	6 (9.2%)	**0.009**
**Blood samples at baseline**			
Red cell distribution width, %	13.4 (12.7 – 14.4)	14.6 (13.7 – 16.3)	** <0.001**
White blood cells, 10^9^/L	6.4 (5.0 – 8.3)	7.7 (5.6 – 10.7)	**0.006**
Neutrophil granulocytes, 10^9^/L	4.7 (3.4 – 6.4)	5.6 (4.1 – 8.9)	**0.001**
Lymphocytes, 10^9^/L	1.01 (0.75 – 1.47)	0.74 (0.58 – 1.12)	** <0.001**
Neutrophil to lymphocyte ratio	4.4 (2.7 – 7.5)	7.23 (4.8 – 12.9)	** <0.001**
C-reactive protein, mg/L	55 (23 – 112)	86 (44 – 130)	**0.001**
Red blood cells, 10^12^/L	4.7 (4.2 – 5.1)	4.4 (3.8 – 4.8)	**0.002**
Hemoglobin, g/dl	13.8 (12.2 – 14.7)	13.0 (11.7 – 13.9)	**0.001**
Platelets, 10^9^/L	194 (155 – 240)	191 (150 – 243)	0.955
Creatinine, mg/dl	1.0 (0.8−1.3)	1.3 (0.9 – 1.8)	** <0.001**
Blood urea nitrogen, mg/dl	17 (13 – 24)	26 (20 – 35)	** <0.001**
Sodium, mmol/L	137 (134 – 139)	137 (135 – 139)	0.423
Potassium, mmol/L	4.0 (3.7 - 4.2)	4.0 (3.8 – 4.4)	0.076
Lactate dehydrogenase, U/L	277 (220 – 369)	327 (231 – 488)	**0.012**

*All values are reported as median and IQR/counts and percentages if not specified otherwise. Bold values represent statisically significant values (P < 0.05)*.

### Clinical Characteristics and Laboratory Findings Stratified by Quartiles of RDW

Patients with higher RDW were older (mean age of 74 ± 15.9 years in Q4 vs. 59 ± 17.1 in Q1) and less frequently male (prevalence of male sex of 48.7% in Q4 vs. 71.6% in Q1). A significant comorbidity burden was noted in patients with increased RDW, with higher rates of arterial hypertension, diabetes mellitus, cardiovascular disease and chronic kidney disease in higher quartiles of RDW. Patients in higher RDW quartiles had lower hemoglobin and red blood cells levels, but higher levels of creatinine and BUN. However, no significant differences were observed across RDW quartiles for concentrations of inflammatory markers, including white blood cells, neutrophil granulocytes, lymphocytes and LDH (Supplementary Table 1).

### Association of RDW With 28-Day Mortality

**Figure 1:**
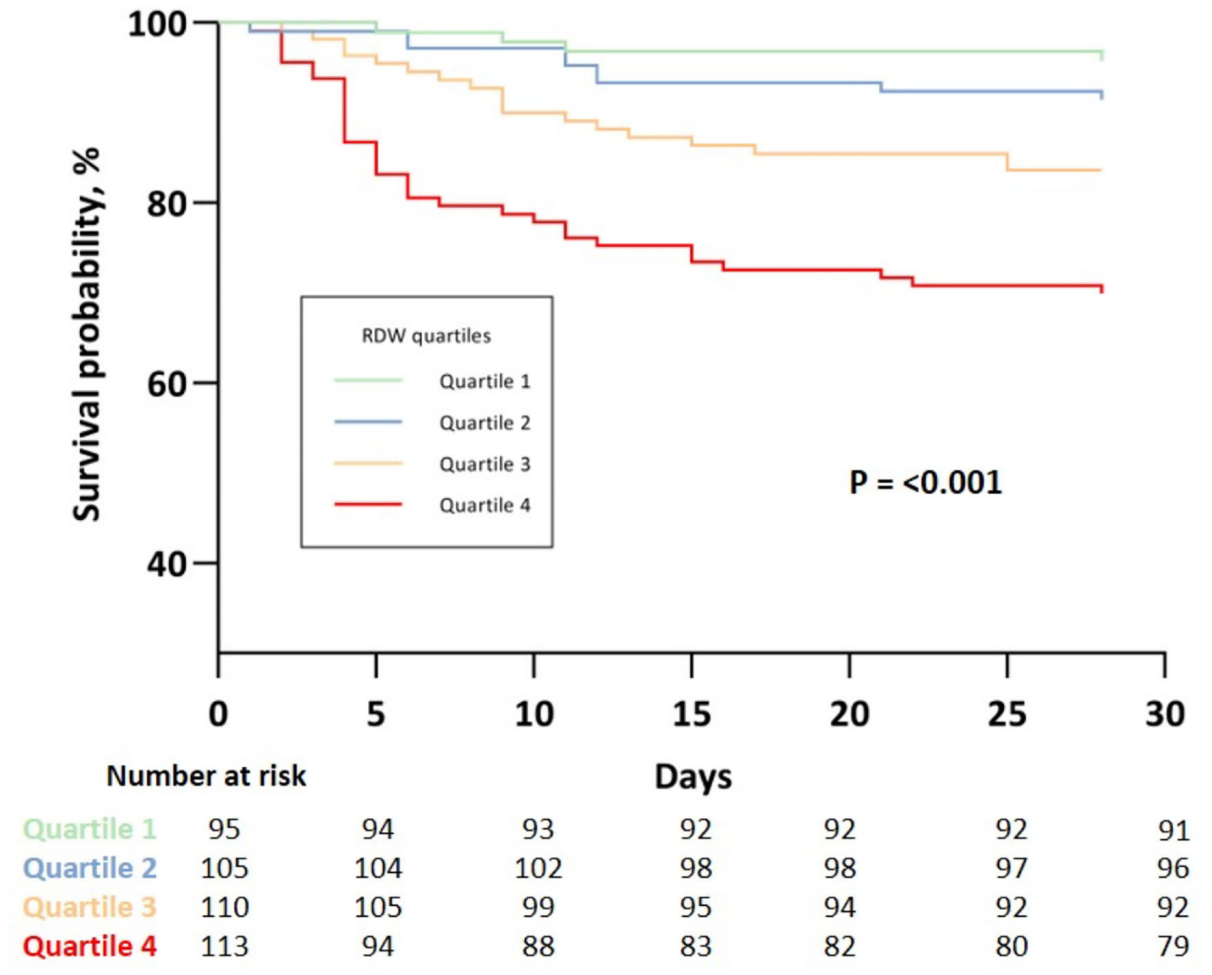
Kaplan Meier analysis of 28-day survival stratified by quartiles of RDW Quartile 1: RDW ≤ 12.7 %; Quartile 2: RDW 12.8 – 13.4 %; Quartile 3: RDW 13.5 – 14.5 %; Quartile 4: RDW ≥ 14.6 %.

Univariable cox regression analysis demonstrated that RDW, either as a continuous or categorical variable at a cut-off level of 14.5%, was significantly associated with 28-day mortality [for RDW on a continuous scale crude hazard ratio (HR) 1.717, 95% confidence interval (CI) 1.462–2.017; *P* < 0.001 and for RDW > 14.5% crude HR 3.426, 95% CI 2.105–5.577; *P* < 0.003]. These results remained statistically significant after multivariable adjustment for clinical confounders (age and gender), comorbidities and laboratory markers associated with prognosis in COVID-19 (Table 2). We also confirmed the prognostic value of RDW for short-term mortality in COVID-19 across several sub-groups, including age, gender, arterial hypertension, history of cardiovascular disease, anemia, increased CRP and creatinine (*P* < 0.05 in all sub-groups) (Figure 1).

**Table 2:** Crude and multivariable cox regression model assessing the impact of RDW on 28-day mortality.

**Statistical model**	RDW – continuous*			**RDW** **>** **14.5 %**		
	**OR**	**95% CI**	**P- value**	**OR**	**95% CI**	**P- value**
Crude model	1.717	1.462 – 2.017	<0.001	3.426	2.105 – 5.577	<0.001
Model 1^a^	1.445	1.224 – 1.706	<0.001	2.412	1.466 – 3.969	0.001
Model 2^b^	1.404	1.181 – 1.669	<0.001	2.230	1.331 – 3.738	0.002
Model 3^c^	1.368	1.126 – 1.662	0.002	1.812	1.060 – 3.096	0.030

*Prior to cox regression analysis laboratory markers were log-transformed*.

a *Model 1: adjusted for age and gender*.

b *Model 2: adjusted for Model 1 + history of arterial hypertension, diabetes mellitus, cardiovascular disease and chronic pulmonary disease*.

c *Model 3: adjusted for Model 1 + Model 2 + hemoglobin, neutrophil to lymphocyte ratio, C-reactive protein, creatinine, lactate dehydrogenase and blood urea nitrogen*.

**Figure 2:**
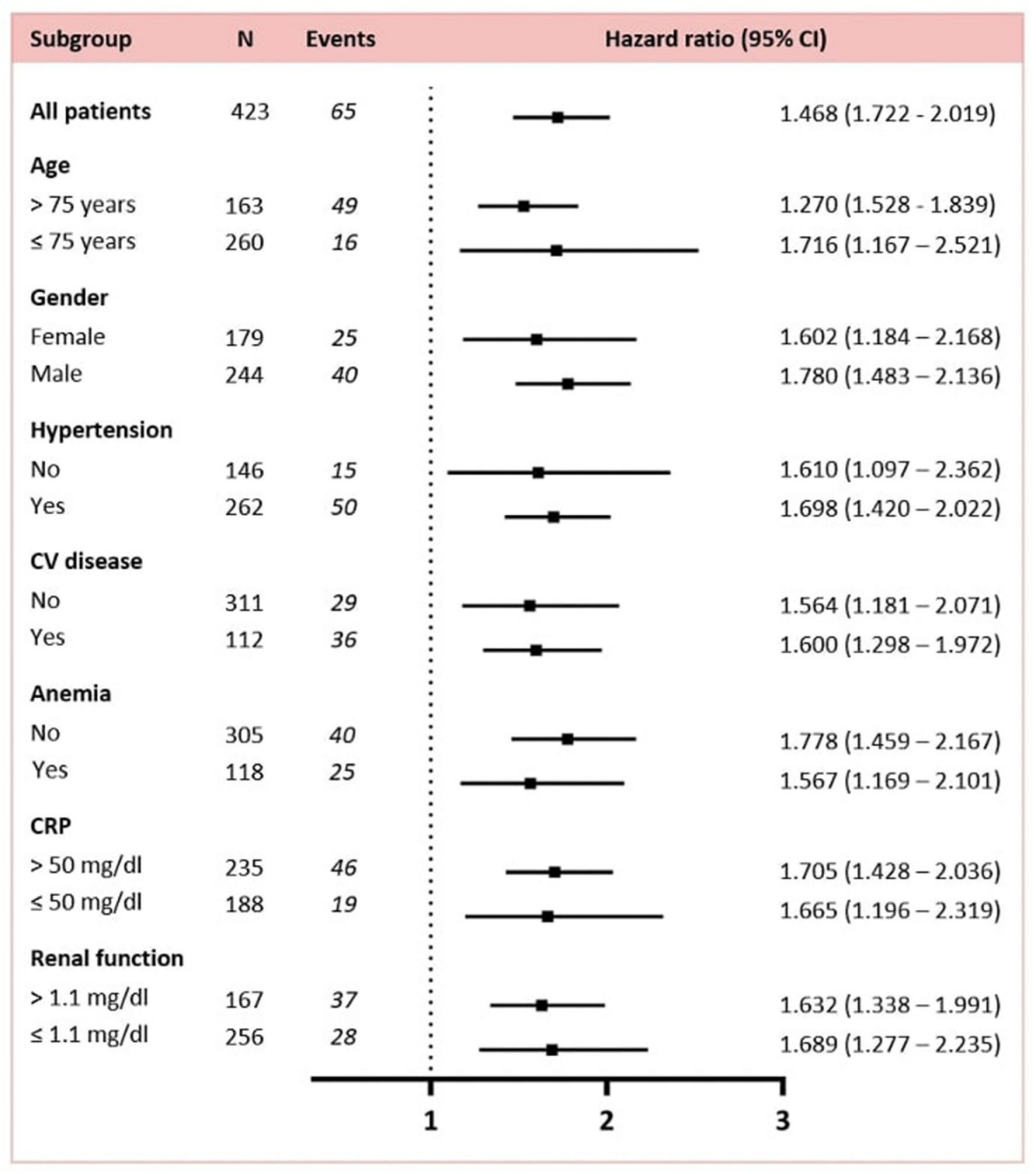
Association of RDW with 28-day mortality in subgroup analysis. Renal function was measured by levels of creatinine upon admission.

Kaplan-Meier estimates showed increasing risk of 28-day mortality with higher quartiles of RDW with log rank test indicating statistically significant differences between the survival curves (*P* < 0.001) (Figure 2).

### Temporal Trends of RDW in the Context of COVID-19

**Figure 3:**
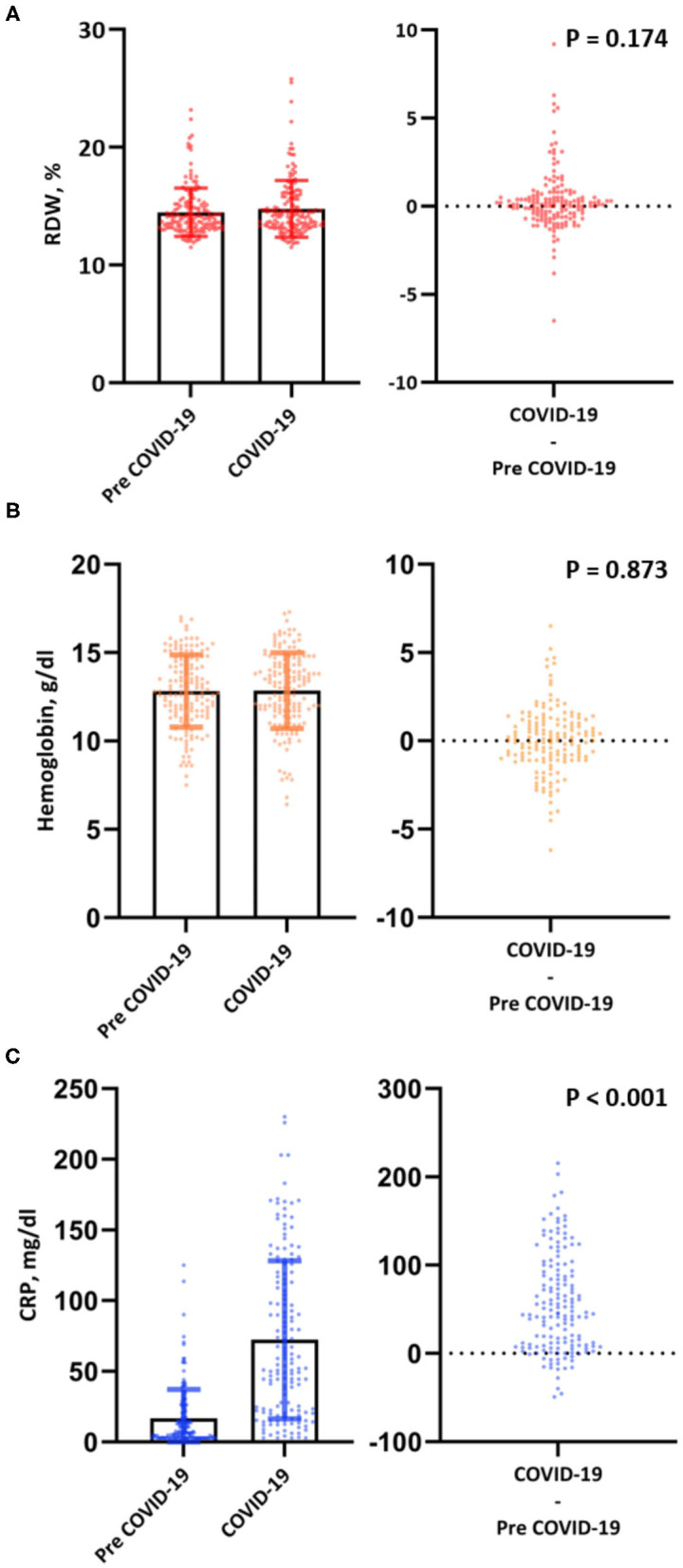
Bar chart with error bars and individual levels/differences plot of RDW **(A)**, hemoglobin **(B)** and CRP **(C)** pre-COVID-19 and at diagnosis for COVID-19.

**Figure 4:**
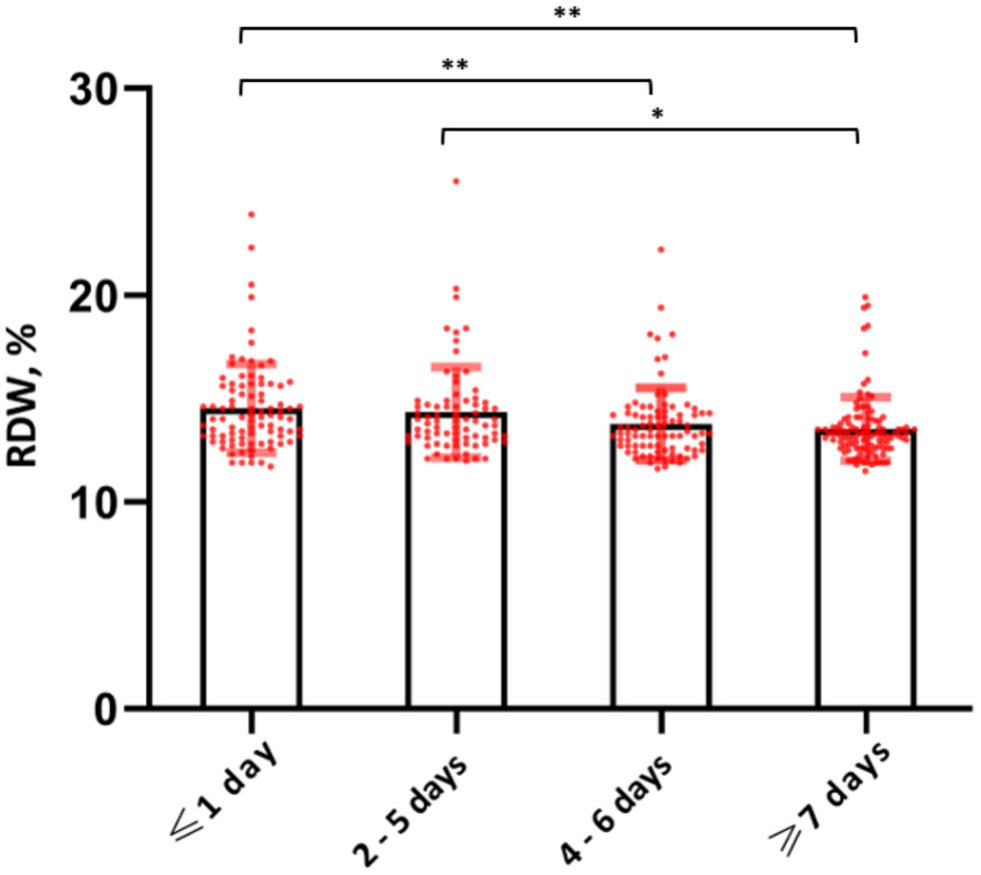
RDW levels stratified by quartiles of symptom onset. *P-value < 0.05; **P-value < 0.01.

Levels of RDW and hemoglobin upon admission for COVID-19 were comparable to pre-COVID-19 levels [14.2% (IQR, 13.3–15.7) vs. 14.0% (IQR, 13.2–15.1) for RDW and 13.1 g/dl (IQR, 11.7–14.4) vs 12.8 g/dl (11.5–14.6) for hemoglobin], while levels of CRP were significantly increased upon admission for COVID-19 [57 mg/dl (IQR, 22–116) vs. 8 mg/dl (3–23)] (Figure 3). Significantly higher levels of RDW were also observed among those presenting early in the disease course of COVID-19, which was defined by patient reported days to onset of symptoms (*P* < 0.001) (Figure 4).

## Discussion

This retrospective, single-center study investigated the prognostic impact of admission levels of RDW in hospitalized patients with COVID-19. The main findings of our study were, that (i) an increased RDW was a significant, independent predictor of 28-day mortality in hospitalized patients with COVID-19, both on a continuous and a categorical level at a prespecified cut-off of > 14.5%, (ii) the association of RDW and short-term mortality persisted across multiple sub-groups, (iii) there was no significant difference between levels of RDW at hospital admission compared to pre-COVID-19 times, and (iv) higher levels of RDW were observed in patients presenting early in the disease course of COVID-19, defined by the time to symptom onset.

### Clinical Evidence

RDW is an easily accessible laboratory marker, that is calculated automatically from complete blood count. It measures the heterogeneity of erythrocyte volumes (anisocytosis) and assesses the variation in size and form of circulating erythrocytes and was thus used almost exclusively for the work-up of anemia. A growing body of evidence has identified RDW a strong negative prognostic marker in a plethora of acute and chronic medical conditions. An association between increased RDW and higher short- and long-term mortality has been established in inflammatory conditions, such as pneumonia (14) or acutely exacerbated COPD (9), cardiovascular disease, such as heart failure (11) or myocardial infarction (22), and in critical illness (7).

Recently it has also been suggested that RDW may be associated with disease severity and mortality in patients with COVID-19 (16–20). Most notably, Foy et al. reported a significantly increased risk of short-term mortality in patients with higher levels of RDW at the time of hospital admission when using a cut-off of 14.5% (21). Lorente and colleagues also found a higher mortality rate in COVID-19 patients with increased RDW—similar to that of established risk scores such as the APPACHE II or SOFA score—who were admitted to an intensive care unit (18). Our study findings further extend the evidence on RDW as a prognostic marker in COVID-19 by demonstrating an increased risk of short-term mortality in a well-defined cohort of 423 hospitalized patients with COVID-19. This association remained statically significant even after adjustment in an extended multivariable regression model, which included several established risk factors for COVID-19. A RDW cut-off of 14.5% has been suggested for prognostication in inflammatory conditions as well as COVID-19 and represented the 4^th^ quartile of RDW in our study population. RDW levels > 14.5% yielded a similar independent, prognostic impact as RDW on a continuous scale.

### Pathophysiologic Mechanisms

While the underlying pathophysiological mechanism linking COVID-19 to RDW has not been elucidated, some theories have emerged offering explanations for the association. Oxidative stress and inflammation have been suggested to alter red cell hemostasis as red blood cell production has been shown to slow down in the context of increased white blood cell and platelet production, which is a common finding in inflammatory conditions such as COVID-19 (23). Inflammatory cytokine release impairs erythrocyte maturation by blocking the activity of erythropoietin, thus causing ineffective red cell production (24). On top of that, inflammation has also been shown to influence bone marrow function and iron metabolism, which in turn lead to an increase of RDW (25–27). Interestingly, we did not observe evidence of increased inflammation in patients with higher RDW values, as levels of white blood cells, lymphocytes, neutrophil granulocytes and CRP were comparable across quartiles of RDW.

Our study findings show that RDW values upon hospital admission for COVID-19 were comparable to previously recorded, pre-COVID-19 levels of RDW. A similar observation was also made for hemoglobin. Hornick et al. also reported a comparable temporal trend of RDW in a cohort of ambulatory and hospitalized patients with COVID-19 (17). To account for the incubation period of COVID-19 we chose a cut-off of at least 14 days from diagnosis for consideration of pre-COVID-19 blood drawings. This is in contrast to the 7 day cut-off used in the study by Hornick and colleagues, which may include some patients in the early stages of SARS-CoV-2 infection (17, 28). We also report pre-COVID-19 levels of CRP – only when measured during the same blood drawing as RDW—which were significantly higher upon admission for COVID-19. While we cannot draw definite conclusions to the circumstances of pre-COVID-19 blood drawings, we can at least demonstrate a substantially lower grade of inflammation compared to blood drawings at admission for COVID-19. Our findings suggest that RDW values upon admission for COVID-19 reflect the individuals' baseline RDW values prior to hospital admission and are independent of the effect of inflammation.

We also stratified levels of RDW according to the patient reported symptom onset of COVID-19 and observed a significant difference across the groups. Patients presenting earlier in the disease course (within 0 or 1 days of symptom onset) had significantly higher levels of RDW compared to those presenting later (> 7 days from symptom onset). As RDW reflects the volume variance of a slow turn-over cell population, changes of RDW usually occur gradually over prolonged periods of time and we should therefore expect RDW levels to be higher in those with a less recent onset of symptoms. Lastly, our study also showed a significantly higher comorbidity burden in those with higher levels of RDW. As RDW has been shown to reflect both acute and chronic illnesses, we may argue that the prognostic value of RDW in the context of COVID-19 is primarily linked to its role as a surrogate marker of general illness in light of our findings (29).

Nevertheless, RDW remained an independent, prognostic marker of short-term mortality in patients with COVID-19 even after adjustment for age, gender, comorbidity burden and established, laboratory risk markers.

## Limitations

A few limitations of our study need to be discussed, most of them inherent to its retrospective design. First, only patients admitted for in-hospital care were included for statistical analysis. Therefore, no extrapolation can be made to patients discharged from the emergency department for ambulatory management. While our study had a fairly large sample size it was conducted only in the Austrian population, which may limit the generalizability of results. Before recommending increased RDW as a robust predictor of worse outcome in COVID-19, further validation in independent populations may be necessary. Third, we only used baseline levels of RDW and hence cannot draw conclusions as to how temporal changes of RDW during hospitalization may affect outcome. Since some studies have shown a prognostic impact of temporal changes of RDW in inflammatory conditions, these results may translate to our population as well. Finally, we only report short-term mortality rates in our population and hence cannot assess the impact of RDW on long-term mortality.

## Conclusions

Our study demonstrates that RDW upon admission is an independent prognostic marker of short-term mortality in hospitalized patients with COVID-19. We did not observe a significant difference between RDW levels at hospital admission for COVID-19 infection compared to pre-COVID-19 blood drawings. RDW may be used as an easily accessible marker to identify COVID-19 patients at risk upon admission and guide adequate clinical management. Further research is needed to confirm our findings.

## Data Availability Statement

The datasets generated for this study are available on request to the corresponding author.

## Ethics Statement

Research involving human subjects complied with all relevant national regulations, institutional policies and is in accordance with the tenets of the Helsinki Declaration (as revised in 2013), and has been approved by the local ethics committee of the city of Vienna (*EK 20-110-VK*).

## Author Contributions

CK, AA, UB, and KH: study concepts, study design, data acquisition, data analysis, and interpretation. CK and AA: statistical analysis. CK, AA, UB, BJ, GA, SE-B, AS, G-CF, MG, PF, and KH: article editing and review. All authors contributed to the article and approved the submitted version after careful revision.

## Conflict of Interest

The authors declare that the research was conducted in the absence of any commercial or financial relationships that could be construed as a potential conflict of interest.
