# Supplementary material for: Red Cell Distribution Width Upon Hospital Admission Predicts Short-Term Mortality in Hospitalized Patients With COVID-19: A Single-Center Experience
Source: Front Med (Lausanne). 2021 Mar 18;8:652707. doi: 10.3389/fmed.2021.652707 (PMC8012506; doi:10.3389/fmed.2021.652707)
Supplement: Supplementary file 1 [file Data_Sheet_1.pdf]

SUPPLEMENTAL MATERIAL

to

Red Cell Distribution Width Upon Hospital Admission Predicts Short-Term Mortality in Hospitalized Patients With COVID-19: A Single-Center Experience

Supplementary figure 1. Flow chart of patients in our retrospective study

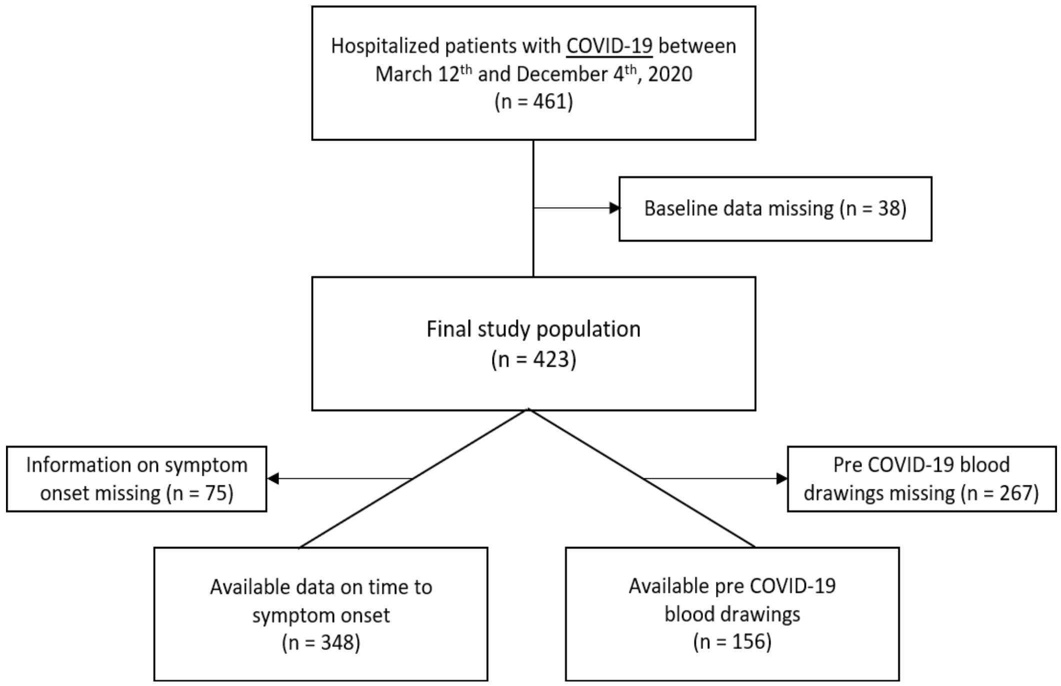

Supplementary table 1. Baseline characteristics of the study population stratified by quartiles of RDW

Characteristics	RDW<br>≤ 12.7 %<br>n = 95	RDW<br>12.8 – 13.4 %<br>n = 105	RDW<br>13.5 – 14.5 %<br>n = 110	RDW<br>≥ 14.6 %<br>n = 113	P-value
**Baseline characteristics**					
Age, years *(mean ± SD)*	59 ± 17.1	65 ± 16.8	71 ± 15.8	74 ± 15.9	**<0.001**
Male sex	68 (71.6%)	67 (63.8%)	54 (49.1%)	55 (48.7%)	0.001
Arterial hypertension	53 (55.8%)	56 (53.3%)	71 (64.5%)	82 (72.6%)	**0.014**
Diabetes mellitus	27 (28.4%)	26 (24.8%)	29 (26.4%)	54 (47.8%)	0.001
Cardiovascular disease	16 (16.8%)	16 (15.2%)	32 (29.1%)	48 (42.5%)	**<0.001**
Chronic pulmonary disease	10 (10.5%)	12 (11.4%)	14 (12.7%)	21 (18.6%)	0.300
Chronic kidney disease	6 (6.3%)	12 (11.4%)	24 (21.8%)	34 (30.4%)	**<0.001**
History of malignancy	6 (6.3%)	13 (12.4%)	17 (15.5%)	19 (16.8%)	**0.120**
**Signs and symptoms**					
Fever	69 (72.6%)	82 (78.1%)	71 (64.5%)	68 (60.2%)	**0.022**
Coughing	54 (56.8%)	57 (54.3%)	58 (52.7%)	39 (34.5%)	**0.003**
Dyspnea	51 (53.7%)	60 (57.1%)	63 (57.3%)	61 (54.0%)	0.922
Gastrointestinal symptoms	27 (38.4%)	24 (22.9%)	23 (20.9%)	17 (15.0%)	0.132
**Blood samples at baseline**					
White blood cells, 10^9/L	6.4 (5.0 – 8.9)	6.4 (5.3 – 7.9)	6.6 (5.0 – 8.3)	6.6 (5.2 – 9.3)	0.793
Neutrophil granulocytes, 10^9/L	4.7 (3.5 – 7.2)	4.7 (3.3 – 5.8)	4.7 (3.5 – 6.7)	5.1 (3.6 – 7.3)	0.493
Lymphocytes, 10^9/L	0.98 (0.74 - 1.43)	1.02 (0.75 – 1.46)	0.99 (0.72 – 1.35)	0.87 (0.60 – 1.46)	0.352
Neutrophil to lymphocyte ratio	4.5 (3.0 – 7.9)	3.8 (2.6 – 6.8)	5.2 (3.1 – 7.5)	5.24 (2.7 – 10.1)	0.126
C-reactive protein, mg/L	65 (24 – 112)	51 (23 – 113)	52 (22 – 107)	79 (29 – 130)	0.157
Red blood cells, 10^{12}/L	4.8 (4.4 – 5.1)	4.7 (4.3 – 5.1)	4.6 (4.0 – 5.0)	4.4 (3.9 – 4.8)	**<0.001**
Hemoglobin, g/dl	14.4 (13.4 – 15.3)	13.8 (13.0 – 14.8)	13.4 (12.0 – 14.6)	12.3 (10.8 – 13.8)	**<0.001**
Platelets, 10^9/L	194 (155 – 233)	200 (157 – 239)	189 (150 – 232)	197 (157 – 266)	0.362
Creatinine, mg/dl	1.0 (0.8 – 1.2)	1.0 (0.8 – 1.2)	1.0 (0.9 – 1.5)	1.2 (0.8 – 1.6)	**0.026**
Blood urea nitrogen, mg/dl	16 (13 – 19)	15 (12 – 24)	18 (13 – 34)	23 (17 – 32)	**<0.001**
Sodium, mmol/L	136 (133 – 138)	137 (134 – 138)	137 (135 – 140)	137 (135 – 140)	**0.023**
Potassium, mmol/L	3.9 (3.7 – 4.2)	4.0 (3.7 – 4.2)	4.0 (3.7 – 4.3)	4.0 (3.8 – 4.4)	0.108
Lactate dehydrogenase, U/L	282 (212 – 380)	270 (222 – 360)	230 (229 – 397)	278 (219 – 386)	0.634
**Hospitalization length, days*** *(mean ± SD)*	12 ± 9.0	15 ± 13.3	13.3 ± 9.2	17 ± 11.6	**0.007**
**28-day mortality**	4 (4.2%)	9 (8.6%)	18 (16.4%)	34 (30.1%)	**<0.001**
